# 急性髓系白血病患者半相合造血干细胞移植后发生急性移植物抗宿主病的危险因素分析

**DOI:** 10.3760/cma.j.cn121090-20250114-00027

**Published:** 2025-10

**Authors:** 丹 冯, 炜 梁, 佳欣 曹, 易耕 曹, 欣 陈, 翠翠 刘, 荣莉 张, 卫华 翟, 嘉璘 魏, 巧玲 马, 栋林 杨, 祎 何, 四洲 冯, 明哲 韩, 爱明 庞, 洪涛 王, 家喜 周, 尔烈 姜

**Affiliations:** 1 中国医学科学院血液病医院（中国医学科学院血液学研究所），血液与健康全国重点实验室，国家血液系统疾病临床医学研究中心，细胞生态海河实验室，天津 300020 State Key Laboratory of Experimental Hematology, National Clinical Research Center for Blood Diseases, Haihe Laboratory of Cell Ecosystem, Institute of Hematology & Blood Diseases Hospital, Chinese Academy of Medical Sciences & Peking Union Medical College, Tianjin 300020, China; 2 天津医学健康研究院，天津 301600 Tianjin Institutes of Health Science, Tianjin 301600, China

**Keywords:** 急性移植物抗宿主病, 白血病，髓系，急性, 造血干细胞移植, Acute graft-versus-host disease, Leukemia, myeloid, acute, Haploidentical transplantation

## Abstract

**目的:**

明确接受单倍体/半相合造血干细胞移植（HID-HSCT）的急性髓系白血病（AML）患者发生急性移植物抗宿主病（aGVHD）的危险因素。

**方法:**

纳入2020年1月至2021年7月于中国医学科学院血液病医院接受HID-HSCT治疗的141例AML患者，aGVHD的累积发生率采用Fine-Gray竞争风险模型进行分析，以复发和死亡作为竞争风险事件，比较不同组之间aGVHD的累积发生率差异。采用Cox比例风险回归模型对潜在危险因素进行单因素及多因素分析，以评估其对aGVHD发生风险的独立影响。

**结果:**

141例患者中男86例（61.0％），女55例（39.0％），患者接受移植的中位年龄为34岁。59例患者在移植后100 d内发生Ⅱ～Ⅳ度aGVHD，86例未发生aGVHD或表现为Ⅰ度aGVHD（0～Ⅰ度aGVHD组）。生存分析显示，Ⅱ～Ⅳ度aGVHD组患者的3年总生存率为68.7％（95％*CI*：57.7％～81.9％），与0～Ⅰ度aGVHD组的78.8％（95％*CI*：70.4％～88.3％）比较差异无统计学意义（*P*＝0.190）。单因素分析显示供者年龄（*P*＝0.020，*HR*＝1.020，95％*CI*: 1.000～1.040）以及女供男性别组合（*P*＝0.033，*HR*＝1.980，95％ *CI*: 1.160～3.380）是Ⅱ～Ⅳ度aGVHD发生的危险因素。多因素分析表明供者年龄（*P*＝0.005，*HR*＝1.026，95％ *CI*: 1.008～1.047）、女供男性别组合（*P*＝0.002，*HR*＝2.339，95％ *CI*: 1.354～4.037）是aGVHD发生的独立危险因素。接受>45岁供者移植的患者在移植后100 d内的Ⅱ～Ⅳ度aGVHD累积发生率显著高于供者年龄≤45岁的患者［54.7％（95％*CI*: 42.3％～67.0％）对31.6％（95％*CI*: 21.0％～42.1％），*P*＝0.006］。女供男性别组合的患者在移植后100 d内的Ⅱ～Ⅳ度aGVHD累积发生率显著高于其他性别组合［56.8％（95％*CI*: 40.4％～73.1％）对36.9％（95％*CI*: 27.5％～46.3％），*P*＝0.015］。

**结论:**

年长供者、女性供者对男性受者是AML患者接受HID-HSCT后发生aGVHD的独立危险因素。

## Abstract (en)

**Objective:**

To identify the risk factors for acute graft-versus-host disease（aGVHD）in patients with acute myeloid leukemia（AML）undergoing haploidentical hematopoietic stem cell transplantation（HID-HSCT）.

**Methods:**

A total of 141 AML patients who underwent HID-HSCT at the Institute of Hematology & Blood Diseases Hospital, Chinese Academy of Medical Sciences, from January 2020 to July 2021 were included. The cumulative incidence of aGVHD was analyzed using the Fine-Gray competing risk model, with relapse and death as competing events, to compare differences between groups. Potential risk factors were evaluated by univariable and multivariable Cox proportional hazards regression analyses to determine their independent effects on aGVHD.

**Results:**

Among the 141 patients, 86（61.0％）were male and 55（39.0％）were female, with a median age at transplantation of 34 years. Within 100 days post-transplant, 59 patients developed grade Ⅱ–Ⅳ aGVHD, whereas 86 patients experienced no or grade Ⅰ aGVHD（the grade 0–Ⅰ aGVHD group）. Survival analysis showed that the 3-year overall survival was 68.7％（95％ *CI*: 57.7％–81.9％）in the grade Ⅱ–Ⅳ aGVHD group, compared with 78.8％（95％ *CI*: 70.4％–88.3％）in the grade 0–Ⅰ aGVHD group, with the difference not being statistically significant（*P*＝0.190）. Univariable analysis identified donor age（*P*＝0.020, *HR*＝1.020, 95％ *CI*: 1.000–1.040）and the female donor–male recipient sex combination（*P*＝0.033, *HR*＝1.980, 95％ *CI*: 1.160–3.380）as risk factors for grade Ⅱ–Ⅳ aGVHD. Multivariable analysis confirmed that donor age（*P*＝0.005, *HR*＝1.026, 95％ *CI*: 1.008–1.047）and the female donor–male recipient sex combination（*P*＝0.002, *HR*＝2.339, 95％ *CI*: 1.354–4.037）were independent risk factors for aGVHD. Patients receiving grafts from donors aged >45 years had a significantly higher 100-day cumulative incidence of grade Ⅱ–Ⅳ aGVHD compared with those receiving grafts from donors ≤45 years［54.7％（95％ *CI*: 42.3％–67.0％）*vs* 31.6％（95％ *CI*: 21.0％–42.1％）, *P*＝0.006］. Similarly, patients with the female donor-male recipient sex combination had a higher 100-day cumulative incidence of grade Ⅱ–Ⅳ aGVHD than those with other sex combinations［56.8％（95％ *CI*: 40.4％–73.1％）*vs* 36.9％（95％ *CI*: 27.5％–46.3％）, *P*＝0.015］.

**Conclusion:**

Older donor age and the female donor-male recipient sex combination remain independent risk factors for aGVHD in patients with AML undergoing HID-HSCT.

异基因造血干细胞移植（allo-HSCT）是治疗急性髓系白血病（AML）的重要策略，特别是对于高风险或者化疗后复发的中低风险患者，HSCT提供了唯一的治愈可能[1]。目前，单倍体/半相合造血干细胞移植（haploidentical HSCT，HID-HSCT）已经取得与全相合HSCT相当甚至更优的疗效，为没有完全相合供者的患者提供了治疗机会，愈发成为部分移植患者的首选[2]–[4]。尽管HID-HSCT可以延长AML患者的生存期，但增加了HSCT后急性移植物抗宿主病（aGVHD）的发生风险，严重影响患者移植预后[4]–[6]。因此，找到aGVHD发生的危险因素并有针对性地预防十分必要。

aGVHD是HSCT后早期主要并发症，研究报道的aGVHD危险因素包括HLA不匹配、供者与受者性别不匹配（如女性供者男性受者）、供者年龄偏大、回输外周血来源的干细胞以及使用包含全身放疗的预处理方案等[7]–[10]。这些研究推进了对aGVHD发生危险因素的认知，但其多在多病种或多种移植方式下进行，缺乏针对特定病种的深入分析。AML作为allo-HSCT最常见的适应证，伴随近年来HID-HSCT的普及应用，其治疗特性和患者免疫背景可能使aGVHD的发生机制与其他病种有所不同。为进一步探讨特定病种的个性化移植策略，我们开展了本项研究，旨在系统评估AML患者接受HID-HSCT后aGVHD的发生及其相关危险因素，为优化移植策略提供科学依据。

## 病例与方法

1. 病例：本研究为回顾性队列研究，共纳入2020年1月至2021年7月期间于中国医学科学院血液病医院接受HID-HSCT的141例AML患者。AML诊断参照2022年WHO第5版诊断标准[11]。AML危险度分层参照2017年版的欧洲白血病网（ELN）指南[12]。

2. 移植及预处理方案：所有患者均接受了来自外周血的HID-HSCT，并采用以白消安+环磷酰胺+抗胸腺细胞球蛋白（BU+CY+ATG）为基础的清髓性预处理（MAC）方案。采用环孢素A（CsA）或他克莫司（FK506）联合短疗程甲氨蝶呤（MTX）方案±霉酚酸酯（MMF）的aGVHD预防方案。

3. 定义：中性粒细胞植入定义为移植后中性粒细胞绝对计数（ANC）≥0.5×10^9^/L，持续3 d；血小板植入定义为移植后血小板计数≥20×10^9^/L，持续7 d且脱离血小板输注。血小板植入不良定义为移植后>28 d未达到血小板植入。aGVHD的诊断标准参照文献[13]。血小板校正计数增量（CCI）计算如下：（PLT_输注后_−PLT_输注前_）×体表面积/血小板输注量。血小板输注无效（PTR）定义为连续两次血小板输注后CCI<4.5×10^9^/L[14]。移植前微小残留病阳性定义为预处理前最后一次骨髓原始细胞流式细胞术检测结果阳性。总生存（OS）时间定义为造血干细胞回输结束至末次随访或死亡的时间。

4. 随访：通过门诊复查、住院病历回顾及电话联系等方式进行随访，随访时间截至移植后3年。

5. 统计学处理：数据分析使用R 4.3.2软件进行。对于分类变量，采用*χ*²检验或Fisher精确检验；对于符合正态分布的连续变量采用“均值±标准差”表示，非正态分布的连续变量采用“中位数（范围）”表示，使用Student's *t*检验或Mann-Whitney *U*检验。使用受试者工作特征曲线（ROC曲线）确定连续变量cut-off值。通过Kaplan-Meier法绘制，其组间比较采用Log-rank检验。采用cmprsk Fine-Gray模型包进行100 d Ⅱ～Ⅳ度aGVHD竞争风险分析，将复发和死亡作为其竞争因素。单因素和多因素分析使用Cox回归模型，并将单因素Cox分析中*P*<0.1的变量及既往报道的危险因素纳入多因素分析。*P*<0.05为差异具有统计学意义。

## 结果

1. 患者临床特征：共纳入141例患者，其中男86例（61.0％），女55例（39.0％），患者接受移植的中位年龄为34岁。在所有患者中，仅有3例接受了非血缘的不全相合移植（9/10）。在移植后100 d内，有59例（41.8％）患者发生了Ⅱ～Ⅳ度aGVHD。根据患者移植后100 d内aGVHD发生情况将患者分为0～Ⅰ度aGVHD组（82例）和Ⅱ～Ⅳ度aGVHD组（59例），两组在移植年龄、性别、疾病危险度分层、移植前疾病状态、移植前微小残留病、供者类型、供受者性别对、供受者血型配对、回输的单个核细胞数和CD34^+^细胞输注量方面差异均无统计学意义（均*P*>0.05）（表1）。Ⅱ～Ⅳ度aGVHD组患者相较0～Ⅰ度aGVHD组患者供者年龄显著偏大［48.0（34.5～55.5）岁对39.5（24.0～49.0）岁，*Z*＝2.406，*P*＝0.013］。两组患者移植后中性粒细胞植入时间、血小板植入时间以及血小板植入不良发生率差异均无统计学意义（均*P*>0.05）。在移植后100 d内，两组分别有1例（1.69％）、3例（3.66％）患者死亡。在0～Ⅰ度aGVHD组患者中观察到2例患者在100 d内复发。对患者随访至移植后3年，35例患者死亡，Ⅱ～Ⅳ度aGVHD组患者的3年OS率为68.7％（95％*CI*：57.7％～81.9％），而0～Ⅰ度aGVHD组患者3年OS率为78.8％（95％*CI*：70.4％～88.3％），两组间差异无统计学意义（*P*＝0.190）（图1）。

**表1:** 接受单倍体/半相合造血干细胞移植的急性髓系白血病患者发生0～Ⅰ度aGVHD组和Ⅱ～Ⅳ度aGVHD组临床特征比较

特征	0～Ⅰ度aGVHD （82例）	Ⅱ～Ⅳ度aGVHD （59例）	统计量	*P*值
性别［例（％）］			1.489	0.222
男	54（65.9）	32（54.2）		
女	28（34.2）	27（45.8）		
移植时中位年龄［岁，*M*（范围）］	37.0（26.3～46.0）	31.0（26.0～39.5）	1.710	0.089
ELN危险度分层［例（％）］		0.496	0.780
低危	25（30.5）	18（30.5）		
中危	36（43.9）	23（39.0）		
高危	21（25.6）	18（30.5）		
初诊血细胞计数［*M*（范围）］				
PLT（×10^9^/L）	47.0（22.0～92.8）	39.0（21.0～79.0）	0.840	0.477
WBC（×10^9^/L）	11.3（3.7～61.8）	11.2（3.0～32.3）	0.277	0.208
HGB（g/L）	84.0（73.0～105.8）	85.5（67.0～100.3）	1.125	0.364
移植前疾病状态［例（％）］		2.957	0.085
CR_1_/CR_2_	69（84.2）	56（94.9）		
PR/NR	13（15.9）	3（5.1）		
移植前微小残留病［例（％）］			0.001	0.978
阴性	57（69.5）	42（71.2）		
阳性	25（30.5）	17（28.8）		
供者类型［例（％）］			0.798	0.372
亲缘	79（96.3）	59（100.0）		
非亲缘	3（3.7）	0（0）		
供受者性别［例（％）］			3.791	0.052
非女供男	66（80.5）	38（64.4）		
女供男	16（19.5）	21（35.6）		
供受者ABO血型［例（％）］			0.001	0.978
相合	57（69.5）	42（71.2）		
主/次不合	25（30.5）	17（28.8）		
aGVHD预防方案［例（％）］		0.009	0.923
CsA+MTX±MMF	38（46.3）	26（44.1）		
FK506+MTX±MMF	44（53.7）	33（55.9）		
MNC回输量［×10^8^/kg，*M*（范围）］	12.1（10.3～15.0）	11.5（10.0～13.3）	1.041	0.253
CD34^+^细胞回输量［×10^6^/kg，*M*（范围）］	3.1（2.5～3.9）	3.0（2.6～3.7）	1.204	0.779
中性粒细胞植入时间［d，*M*（范围）］	12.0（11.0～15.0）	12.0（11.0～14.5）	0.884	0.420
血小板植入时间［d，*M*（范围）］	16.0（13.0～23.0）	15.0（13.0～21.3）	0.840	0.443
30 d血小板输注无效［例（％）］	26（31.7）	16（27.1）	0.161	0.688
血小板植入不良［例（％）］	12（14.6）	6（10.2）	0.279	0.598
100 d内复发［例（％）］	2（2.4）	0（0）		0.510
100 d内死亡［例（％）］	3（3.7）	1（1.7）		0.640

**注** aGVHD：急性移植物抗宿主病；ELN：欧洲白血病网；CR_1_/CR_2_：第1、2次完全缓解；PR：部分缓解；NR：未缓解；CsA：环孢素A；MTX：甲氨蝶呤；MMF：霉酚酸酯；FK506：他克莫司；MNC：单个核细胞

**图1:**
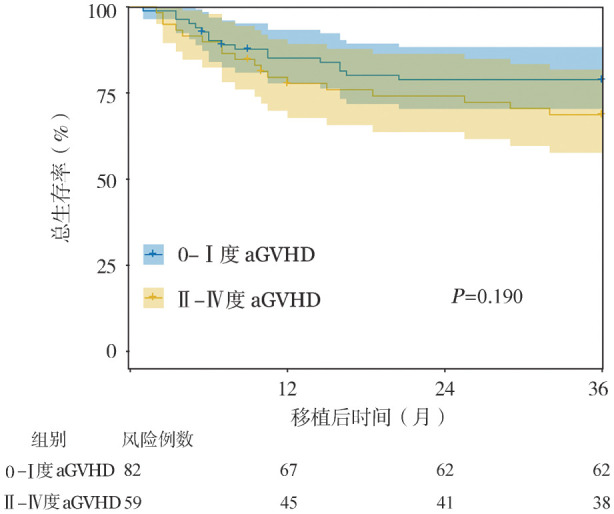
141例接受单倍体/半相合造血干细胞移植的急性髓系白血病患者总生存分析

2. 接受HID-HSCT的AML患者发生Ⅱ～Ⅳ度aGVHD的风险因素分析：在单因素Cox模型中，移植前疾病状态、供者年龄、供受者性别、aGVHD预防方案、MNC回输量、CD34^+^细胞回输量、是否血小板植入不良等因素被纳入分析，结果显示供者年龄（*P*＝0.020，*HR*＝1.020，95％ *CI*: 1.000～1.040）以及女供男性别组合（*P*＝0.033，*HR*＝1.980，95％ *CI*: 1.160～3.380）是Ⅱ～Ⅳ度aGVHD发生的危险因素（表2）。

**表2:** 接受单倍体/半相合造血干细胞移植的急性髓系白血病患者发生Ⅱ～Ⅳ度aGVHD风险因素的单因素Cox回归分析

影响因素	单因素分析	
	*P*值	*HR*（95％*CI*）
供者年龄	0.020	1.020（1.000～1.040）
移植前疾病状态	0.082	0.357（0.112～1.140）
女供男性别组合	0.033	1.980（1.160～3.380）
MNC回输量（×10^8^/L）	0.313	0.965（0.899～1.030）
CD34^+^细胞回输量（×10^6^/L）	0.259	0.894（0.736～1.090）
包含FK506的aGVHD预防方案	0.921	1.030（0.614～1.720）
血小板植入不良	0.473	0.734（0.315～1.710）

**注** aGVHD：急性移植物抗宿主病；MNC：单个核细胞；FK506：他克莫司

将单因素分析中*P*<0.1的因素及既往报道的aGVHD的危险因素纳入多因素分析，以双侧*P*<0.05为差异有统计学意义。结果显示，在多因素Cox回归分析中（表3），供者年龄（*P*＝0.005，*HR*＝1.026，95％ *CI*: 1.008～1.047）依然是aGVHD发生的独立风险因素，供者每增加1岁，患者发生aGVHD的风险增加2.6％。此外，女供男性别组合（*P*＝0.002，*HR*＝2.339，95％ *CI*: 1.354～4.037）也是aGVHD发生的独立危险因素，进一步支持了女性供者在男性受者中的移植可能增加aGVHD发生的风险。而移植前疾病状态（*P*＝0.107，*HR*＝0.384，95％ *CI*: 0.120～1.230）不是影响aGVHD发生的风险因素。

**表3:** 接受单倍体/半相合造血干细胞移植的急性髓系白血病患者发生Ⅱ～Ⅳ度aGVHD风险因素的多因素Cox回归分析

影响因素	多因素分析	
	*P*值	*HR*（95％*CI*）
供者年龄	0.005	1.026（1.008～1.047）
移植前疾病状态	0.107	0.384（0.120～1.230）
女供男性别组合	0.002	2.339（1.354～4.037）
MNC回输量（×10^8^/L）	0.336	0.965（0.897～1.038）
CD34^+^细胞回输量（×10^6^/L）	0.472	0.924（1.082～0.746）

**注** aGVHD：急性移植物抗宿主病；MNC：单个核细胞

3. 老年供者、女供男模式以及细胞回输量对Ⅱ～Ⅳ度aGVHD发生的影响：基于ROC曲线分析，我们确定了供者年龄的最佳界值为45岁，并将供者分为年龄>45岁和≤45岁两组。Fine-Gray检验结果表明，接受年龄>45岁供者外周血干细胞的患者，在移植后100 d内的Ⅱ～Ⅳ度aGVHD累积发生率［54.7％（95％*CI*：42.3％～67.0％）］显著高于接受年龄≤45岁供者的患者［31.6％（95％*CI*：21.0％～42.1％）］（*P*＝0.006）（图2A）。此外，女供男性别组合的患者在移植后100 d内的Ⅱ～Ⅳ度aGVHD累积发生率为56.8％（95％*CI*：40.4％～73.1％），显著高于非女供男组的36.9％（95％*CI*：27.5％～46.3％）（*P*＝0.015）（图2B）。提示供者年龄和性别匹配模式在aGVHD的预防中具有重要的参考价值。

尽管单因素和多因素分析结果并未显示细胞回输量与Ⅱ～Ⅳ度aGVHD之间存在显著的相关性，但由于样本量有限，不能完全排除潜在影响。通过ROC曲线确定MNC回输量的最佳界值为13.5×10⁸/L，竞争风险分析结果显示，MNC回输量低于13.5×10⁸/L的患者组，其Ⅱ～Ⅳ度aGVHD的发生率显著高于MNC回输量≥13.5×10⁸/L的患者组［48.9％（95％*CI*：38.7％～59.1％）对28.3％（95％*CI*：15.1％～41.4％），*P*＝0.024］（图2C），提示较低的MNC回输量可能与重度aGVHD的发生存在一定关联。另一方面，依据CD34^+^细胞回输量的最佳界值4.3×10⁶/L将患者分为两组，但两组之间在Ⅱ～Ⅳ度aGVHD发生率方面差异并无统计学意义［45.2％（95％*CI*：36.1％～54.4％）对26.9％（95％*CI*：9.5％～44.4％），*P*＝0.087］（图2D）。表明，尽管MNC回输量可能与aGVHD的发生存在一定的关联性，但CD34^+^细胞回输量对aGVHD发生的影响仍需进一步研究来明确。

**图2:**
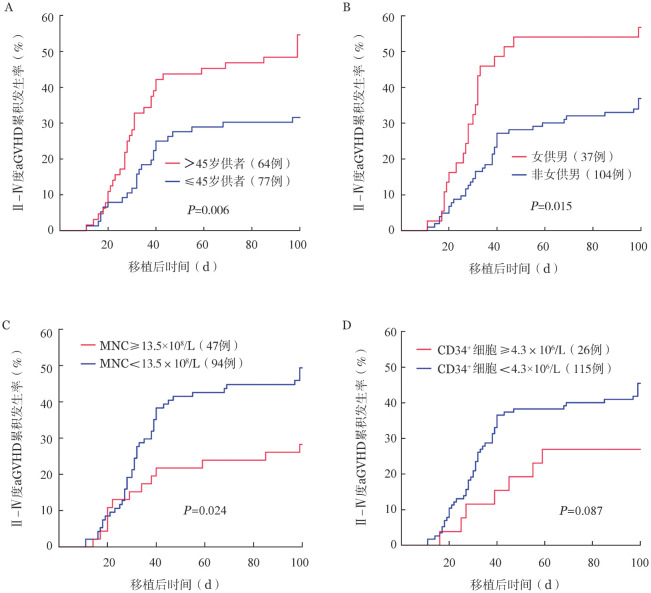
接受单倍体/半相合造血干细胞移植的急性髓系白血病患者发生Ⅱ～Ⅳ度急性移植物抗宿主病（aGVHD）的竞争风险分析（以复发和死亡为其竞争因素） **A** 供者年龄；**B** 供受者性别组合；**C** 单个核细胞（MNC）回输量；**D** CD34^+^细胞回输量

4. 亚组分析：我们进一步探究接受不同的aGVHD预防方案的患者，是否存在不同的Ⅱ～Ⅳ度aGVHD发生的独立危险因素。我们分别对接受CsA和FK506的两组患者进行风险因素分析。单因素分析结果显示，在接受CsA预防方案的患者队列中，供者年龄以及女供男性别组合仍是该部分患者发生Ⅱ～Ⅳ度GVHD的危险因素（*P*<0.05），多因素分析显示,供者年龄是接受CsA预防方案的患者发生Ⅱ～Ⅳ度aGVHD的独立危险因素（*P*＝0.047，*HR*＝1.030，95％ *CI*: 1.000～1.060）。竞争风险分析显示，接受年龄>45岁供者移植的患者有更高的Ⅱ～Ⅳ度aGVHD累积发生率［57.1％（95％*CI*：38.3％～76.0％）对27.8％（95％*CI*：12.9％～42.7％），*P*＝0.015］。而在针对接受FK506预防方案的患者危险因素分析中，未能发现该部分患者发生Ⅱ～Ⅳ度aGVHD的危险因素。

## 讨论

近年来，多项研究在不同的疾病模式以及移植模式下提出了年长供者、女性供者对男性受者是aGVHD发生的危险因素。本研究对单一病种的限定移植模式展开研究，证实了供者年龄大于45岁、女供男性别组合是AML患者接受HID-HSCT发生aGVHD的独立危险因素。

aGVHD作为allo-HSCT后早期主要并发症，其发生率随着诊疗技术的进步呈降低趋势。2023年一项由刘启发教授团队开展的前瞻性多中心研究表明，在接受HID和全相合（MSD）移植的患者中，Ⅱ～Ⅳ度aGVHD的总体累积发生率分别为40％和46％（*P*＝0.848）[15]。在另一项更早期的针对急性淋巴细胞白血病（ALL）患者接受allo-HSCT的研究也显示了相似的结果[16]。本研究中患者的Ⅱ～Ⅳ度aGVHD发生率为41.8％，与既往的报道结果一致。

供者年龄是影响aGVHD发生的重要因素。Kim等[17]在一项针对204例老年AML患者接受移植治疗的研究中指出，供者年龄超过35岁显著增加了≥60岁的老年患者移植后发生aGVHD的风险。这一结果与本研究的结论相符。这一现象可能与年长供者免疫细胞功能的衰退以及与炎症相关的免疫反应增强有关,但目前仍不清楚哪些与衰老相关的细胞或亚细胞变化导致了年龄较大供者的负面影响。Sanz等[18]开展了一项基于1 011例完全缓解的AML患者的研究，认为患者可以从<30岁的供者干细胞中获益。需要注意的是，本研究基于ROC曲线将供者年龄的界值设定为45岁，显著高于上述研究中设定的35岁，这可能与本研究患者的中位年龄较小（34岁对≥60岁）有关。尽管此前在Leukemia杂志发表的一项研究中[8]，作者纳入了高风险的AML及ALL患者，将供者年龄>40岁且受者年龄<30岁作为独立的年龄组别进行分析，结果表明该种年龄分组不是重度aGVHD发生的危险因素。这提示评估供者年龄的最佳阈值可能因受者年龄的不同而有所调整，并且有必要在限定病种/移植模式下开展，需要在未来研究中进一步探索。

供者性别与性别错配在本研究中也被认为是aGVHD的高危因素,这与先前的报告一致[2],[8]。一项包含1 193份供体-受体样本的基因组研究发现，4个基因（PCDH11Y、USP9Y、UTY和NLGN4Y）中Y染色体编码的单核苷酸多态性与女性供者男性患者的aGVHD有关[19]，这种旁系同源X-Y错配解释了男性受者与女性供者的aGVHD风险。

在本研究中，未能证实移植前肿瘤负荷、aGVHD预处理方案、血小板植入等因素是aGVHD发生的风险因素，这可能是来自单中心数据导致，也可能是在限定疾病及限定移植方式下的结果。在此前已公开的诸多研究中，这些因素也同样存在不确定性[20]。尽管因素分析没有显示MNC和CD34^+^细胞回输量是影响aGVHD发生的因素，但竞争风险分析显示MNC回输量可能是影响aGVHD发生的一个重要因素，而CD34^+^细胞回输量的作用尚不明确，因此，未来应通过多中心的大样本量研究进一步验证这些结论。其次，aGVHD的发生不仅与供者年龄和性别相关，还可能与基因多样性、T细胞重排以及免疫抑制治疗的选择等多种因素密切相关。因此，进一步探索免疫学标志物、T细胞谱系以及遗传背景对GVHD的影响将有助于更全面地理解其发生机制，并为个体化治疗提供依据。

综上所述，本研究发现年长供者和女供男性别组合是AML患者接受HID-HSCT后发生aGVHD的独立危险因素。这些结果为临床接受HID-HSCT治疗的AML患者的供者选择提供了证据支持，并为优化GVHD的预防与治疗策略提供了重要的临床依据。
